# Hepatobiliary Phase Features of Preoperative Gadobenate-Enhanced MR can Predict Early Recurrence of Hepatocellular Carcinoma in Patients Who Underwent Anatomical Hepatectomy

**DOI:** 10.3389/fonc.2022.862967

**Published:** 2022-08-03

**Authors:** Wanmin Liu, Kairong Song, Wei Zheng, Lei Huo, Sisi Zhang, Xiaowen Xu, Peijun Wang, Ningyang Jia

**Affiliations:** ^1^ Department of Radiology, Tongji Hospital, School of Medicine, Tongji University, Shanghai, China; ^2^ Department of Radiology, Eastern Hepatobiliary Surgery Hospital, The Third Affiliated Hospital of Shanghai Naval Military Medical University, Shanghai, China

**Keywords:** gadobenate dimeglumine, magnetic resonance imaging, hepatocellular carcinoma, early recurrence, anatomical hepatectomy

## Abstract

**Purpose:**

The purpose of this study was to establish a model for predicting early recurrence (≤2 years) of hepatocellular carcinoma (HCC) after anatomical hepatectomy based on the hepatobiliary phase (HBP) imaging characteristics of gadobenate-enhanced MRI.

**Methods:**

A total of 155 patients who underwent anatomical hepatectomy HCC therapy and gadobenate-enhanced MRI were included retrospectively. The patients were divided into the early recurrence-free group (*n* = 103) and the early recurrence group (*n* = 52). Univariate and multivariate Cox regression analysis was used to determine the independent risk factors related to early recurrence, and four models were established. The preoperative model with/without HBP imaging features (HBP-pre/No HBP-pre model) and the postoperative model with/without HBP imaging features (HBP-post/No HBP-post model). Bootstrap resampling 1,000 times was used to verify the model and displayed by nomograms. The performance of nomograms was evaluated by discrimination, calibration, and clinical utility. Net reclassification improvement (NRI) and integrated discrimination improvement (IDI) were used to evaluate the differences between models and to select the optimal model.

**Results:**

Shape, arterial peritumoral enhancement, AFP-L3, and peritumoral hypointensity on HBP were identified as independent risk factors. Prothrombin time (PT) and r-glutamyltransferase (GGT) were selected by multivariate Cox regression. These six factors construct the HBP-pre model. Removing peritumoral hypointensity on HBP was the No HBP-pre model. Adding microvascular invasion (MVI) and microscopic capsule factors were the HBP-post and No HBP-post model. The C-index was 0.766, 0.738, 0.770, and 0.742, respectively. The NRI and IDI of the HBP-pre vs. the No HBP-pre model and the HBP-post vs. the No HBP-post model significantly increased 0.258, 0.092, 0.280, and 0.086, respectively. The calibration curve and decision curve analysis (DCA) had good consistency and clinical utility. However, the NRI and IDI of the No HBP-post vs. the No HBP-pre model and the HBP-post vs. the HBP-pre model did not increase significantly.

**Conclusions:**

Preoperative gadobenate-enhanced MR HBP imaging features significantly improve the model performance while the postoperative pathological factors do not. Therefore, the HBP-pre model is selected as the optimal model. The strong performance of this model may help hepatologists to assess the risk of recurrence in order to guide the selection of treatment options.

## Introduction

Hepatocellular carcinoma (HCC) is the fourth leading cause of cancer-related death worldwide (1) and the second most common cause of years of life lost due to cancer worldwide after lung cancer between 2005 and 2015 (2). Surgical resection is the best therapy for treatment of HCC patients with well-preserved liver function and isolated tumors (3). Although surgical resection has a good long-term survival, 50%–70% of HCC patients still develop tumor recurrence within 5 years (4, 5). The early recurrence (within 2 years), considered a “real recurrence”, is now recognized as an important factor with a poor prognosis (6, 7).

Early recurrence (within 2 years) is associated with the aggressive pathological factors, such as microvascular invasion (MVI), microsatellite nodules, and worse differentiation (6–9). Therefore, although early recurrence is critical in treatment, outcome, and prognosis, it is mostly evaluated by postoperative pathological examination, and its application in the preoperative context is still limited (10).

Gadobenate dimeglumine (Gd-BOPTA) is a hepatobiliary-specific agent (11–13). This agent not only can be used to obtain dynamic contrast-enhanced (DCE) MRI of the liver but also can obtain the specific imaging of hepatobiliary phase (HBP) within 40 to 120 min after injection (14). Most of the preoperative prediction models of early recurrence after hepatectomy with MRI features are traditional extracellular contrast agent MR or gadoxetic acid-enhanced MR (10, 15–17). Traditional extracellular contrast agents have no HBP, and gadoxetic acid-enhanced MR does not have a real delayed phase (DP), but a transitional phase (TP) instead. In this study, gadobenate-enhanced MR with real DP and HBP can explore the role of HBP imaging features in predicting the early recurrence of HCC and, at the same time, can be compared with DCE MRI.

The literature on the application of gadobenate-enhanced MR in the liver was reviewed. There are literatures using gadobenate-enhanced MR to evaluate the prediction accuracy of MVI in HCC patients (14), the prediction value of pathological grade for HCC (18), the clinical value of HBP hypointensity in the diagnosis of HCC (12), the distinction between small HCC and dysplastic nodules (DNs) (19), and the diagnostic value of hepatitis and hepatic fibrosis (20). However, it has not been found to predict the early recurrence of HCC. This study aims to explore features and optimized models from the HBP phase of Gd-BOPTA-enhanced MR in predicting the early recurrence of HCC.

## Materials and methods

### Patients

This study was approved by the Ethics Committee of Eastern Hepatobiliary Surgery Hospital, the Third Affiliated Hospital of Shanghai Naval Military Medical University, China, and waived the requirement of obtaining written informed consent.

**Figure 1:**
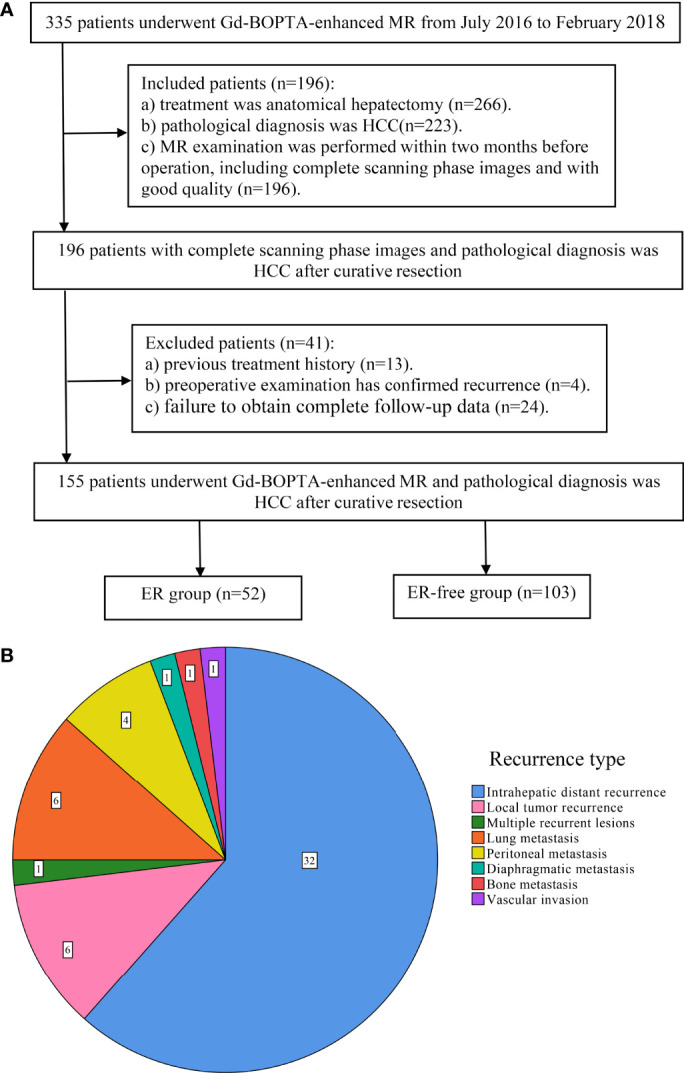
Flowchart of study population and the type of early recurrence. **(A)** The workflow of patient selection for this study; **(B)** pie chart of early recurrence type.

A total of 335 patients who underwent Gd-BOPTA-enhanced MR examination in the Eastern Hepatobiliary Surgery Hospital from July 2016 to February 2018 were preliminarily screened. The inclusion criteria were as follows: (a) the treatment was anatomical hepatectomy and pathological diagnosis was HCC; and (b) Gd-BOPTA-enhanced MR examination was performed within 2 months before operation, including complete scanning phase images (arterial phase, portal phase, DP, and HBP) with good quality. The exclusion criteria were as follows: (a) previous treatment history of HCC, such as surgery, transarterial chemoembolization, local ablation, systemic chemotherapy, or radiotherapy; (b) preoperative examination has confirmed recurrence; and (c) failure to obtain complete follow-up data. A total of 155 patients participated in the study (mean age 55.41 ± 10.584, 133 male patients). The patient screening flowchart is shown in **Figure 1A**.

### MR Image Acquisition

All MR images were obtained from GE Optima MR360 1.5 T equipped with 8-channel abdominal coils. Patients were fasted for 4 h before the scan. Gd-BOPTA (MultiHance, Bracco) with a total dose of 0.1 mmol/kg was injected into the median cubitus vein at a rate of 2.0 ml/s with a high-pressure syringe, followed by washing with 20 ml of normal saline. Arterial, portal, delayed, and hepatobiliary scans were performed at 20–30 s, 50–60 s, 90–120 s, and 60 min after the injection of GD-BOPTA, respectively. HBP scans were performed at 120 min after the injection of contrast agent for patients with impaired liver function. Fat-suppressed T2-weighted images, T1WI, and FIESTA were collected. Detailed scanner and scan parameters can be found in **Supplementary Table E1**.

### MRI Analysis

All MR images were blindly reviewed by two radiologists with more than 10 years of experience in liver MRI. In case of disagreement, a third radiologist (with more than 10 years of experience in liver MRI) was employed to help resolve the issue. Two radiologists evaluated 10 imaging features defined in LI-RADS v2018 (21): (a) MRI tumor diameter, (b) blood products in mass, (c) radiological capsule enhancement, (d) mild-moderate T2 hyperintensity, (e) restricted diffusion, (f) nonrim arterial phase hyperenhancement (APHE), (g) rim APHE, (h) nonperipheral “washout”, (i) HBP hypointensity, and (j) delayed central enhancement. The definition of LI-RADS features can be found in **Supplementary Table E2**.

Ten non-LI-RADS imaging features were also evaluated: (a) tumor number: only one lesion was solitary, and two or more lesions were multiple; (b) shape: round- or oval-like were defined as regular, while others are defined as irregular, such as lobulated, star awn, and needle-like; (c) margin: nodular tumors with smooth boundary are smooth margin, and non-nodular tumors with irregular contour and budding into the surrounding liver parenchyma can be seen as non-smooth margin (22, 23); (d) intratumoral necrosis: the area in the tumor without enhancement, high signal on T2WI, low signal on T1WI; (e) enhancement pattern: typical enhancement meets the “wash in and wash out” enhancement, and the rest are typical; (f) arterial peritumoral enhancement: defined as the enhancement outside the tumor boundary in the late stage of arterial phase or early stage of portal phase, extensive contact with the tumor edge, which becomes isointense during the DP (23–25); (g) peritumoral hypointensity on HBP: presenting as hypointense areas of liver parenchyma around the boundary of tumor in crescent shape, wedge shape, or flame-like shape on HBP images (14, 26); (h) MRI liver cirrhosis: defined as irregular, nodular, or shrinking liver, and signs of ascites or formation of collateral circulation of decompensated portal system (27); (i) splenomegaly; and (j) ascites.

### Follow-Up Surveillance

At the second month after anatomical hepatectomy, serum alpha-fetoprotein (AFP) level, liver function tests, chest CT, and dynamic contrast-enhanced CT or MR of the abdomen were performed every 3 months during the 2-year follow-up period. Early recurrence was defined as local tumor recurrence (a single recurrent lesion located near the surgical margin), intrahepatic distant recurrence (a single recurrent lesion located in the remnant liver except the surgical margin), and multiple recurrent lesions of the remnant liver or extrahepatic metastasis within 2 years after surgical resection (**Figure 1B**). Recurrence-free survival (RFS) was defined as the period from the date of surgery to the date of first progression, recurrence, metastasis, or the end of follow-up.

### Model Development, Evaluation, and Comparison

We established four models to predict early recurrence of HCC. Two preoperative models: the No HBP-pre model and the HBP-pre model, including clinical and imaging features. After adding the postoperative pathological factors, two postoperative models were established: the No HBP-post model and the HBP-post model. The four models are shown by nomograms. The discrimination of nomogram was evaluated by Harrell’s concordance index (C-index) (28, 29). The predictive accuracy of the nomogram was validated by calibration curves obtained by 1,000 bootstrap examples (29). Decision curve analysis (DCA) was conducted to determine the clinical utility of the model by quantifying the net benefits at different threshold probabilities. ANOVA was used to compare whether the differences between models were statistically significant. NRI and IDI were used to compare the diagnostic accuracy improvement level and overall improvement level between models (30). After the optimal model is finally selected, the patients are divided into two groups (low-risk and high-risk groups) according to the risk score of the model, and the early recurrence rate is calculated.

### Statistical Analysis

Continuous variables conforming to normal distribution and homogeneity of variance are represented by means ± standard deviations (SD) and were compared using Student’s *t*-test, and inconsistent continuous variables are represented by median (range) and compared with the Mann–Whitney *U* test. Categorical variables were compared using the *χ* ^2^ test. The RFS probability was estimated by the Kaplan–Meier method and compared by the log-rank test. The radiological and pathological factors with *p* < 0.1 and the clinical factors with *p* < 0.3 in univariate Cox regression analysis (*p* = 0.304 of PT was also included) were included in multivariate Cox regression analysis (forward LR) to establish the model. All analyses were performed with SPSS software (version 25.90, IBM) and R software ( version 3.6.0, http://www.r-project.org).

## Results

### Baseline Patient Characteristics

The comparison of main clinical, radiological, and pathological factors is shown in **Table 1**. The comparison of all baseline data can be found in **Supplementary Table E3**.

**Table 1:** Baseline patient characteristics.

Characteristic	Total (*n* = 155)	ER-free (*n* = 103)	ER (*n* = 52)	*p*-value
**Clinical features**				
Age	55.41 ± 10.584	55.55 ± 10.117	55.12 ± 11.552	0.809
Sex				
Male	133 (85.8%)	90 (87.4%)	43 (82.7%)	0.430
Female	22 (14.2%)	13 (12.6%)	9 (17.3%)	
BCLC stage				
0	38 (24.5%)	29 (28.2%)	9 (17.3%)	0.330
A	104 (67.1%)	66 (64.1%)	38 (73.1%)	
B	13 (8.4%)	8 (7.8%)	5 (9.6%)	
Liver disease				
HBV	139 (89.7%)	92 (89.3%)	47 (90.4%)	0.837
None or other	16 (10.3%)	11 (10.7%)	5 (9.6%)	
AFP-L3				
Negative	104 (67.1%)	76 (73.8%)	28 (53.8%)	**0.013**
Positive	51 (32.9%)	27 (26.2%)	24 (46.2%)	
AFP (ng/L)	25 (4.4–175.7)	16.8 (4.5–189)	44.2 (3.6–167.2)	0.417
PIVKA-II (mAU/ml)	119 (30–916)	119 (27–883)	120 (47–1335.25)	0.381
GLOB (g/L)	26.2 (23.2–29)	26.75 (23.875–26.75)	25.2 (22.4–25.2)	0.056
GGT (U/L)	40 (26–73)	39 (25–63)	43 (28.5–102.25)	0.212
AFU (U/L)	23 (19–28)	23 (18–27)	23.5 (19.25–29.75)	0.193
PT (S)	12 (11.4–12.7)	12.1 (11.4–12.8)	11.9 (11.2–12.575)	0.176
CHOL (mmol/L)	3.94 (3.47–4.32)	4.0234 (3.55–4.38)	3.8177 (3.375–4.0825)	0.155
**Pathologic factors**				
MVI				
Absent	105 (67.7%)	75 (72.8%)	30 (58.8%)	0.057
Present	50 (32.3%)	28 (27.2%)	22 (42.3%)	
Microscopic capsule				
Present	130 (83.9%)	90 (87.4%)	40 (76.9%)	0.095
Absent	25 (16.1%)	13 (12.6%)	12 (23.1%)	
**MRI features**				
MRI tumor diameter (cm)	3 (2.1–4.3)	2.9 (2.1–4.1)	3.1 (2.2–5.175)	0.221
Tumor number				
Solitary	138 (89%)	94 (91.3%)	44 (84.6%)	0.211
Multiple	17 (11%)	9 (8.7%)	8 (15.4%)	
Shape				
Regular	96 (61.9%)	74 (71.8%)	22 (42.3%)	**<0.001**
Irregular	59 (38.1%)	29 (28.2%)	30 (57.7%)	
Margin				
Smooth	82 (52.9%)	63 (61.2%)	19 (36.5%)	**0.004**
Non-smooth	73 (47.1%)	40 (38.8%)	33 (63.5%)	
Intratumoral necrosis				
Absent	120 (77.4%)	83 (80.6%)	37 (71.2%)	0.185
Present	35 (22.6%)	20 (19.4%)	15 (28.8%)	
Radiological capsule enhancement				
Complete	60 (38.7%)	50 (48.5%)	10 (19.2%)	**0.002**
Incomplete	74 (47.7%)	41 (39.8%)	33 (63.5%)	
Absent	21 (13.5%)	12 (11.7%)	9 (17.3%)	
Nonrim APHE				
Present	104 (67.1%)	78 (75.7%)	26 (50.0%)	**0.001**
Absent	51 (32.9%)	25 (24.3%)	26 (50.0%)	
Rim APHE				
Absent	115 (74.2%)	82 (79.6%)	33 (63.5%)	**0.030**
Present	40 (25.8%)	21 (20.4%)	19 (36.5%)	
Nonperipheral “washout”				
Present	101 (65.2%)	72 (69.9%)	29 (55.8%)	0.081
Absent	54 (34.8%)	31 (30.1%)	23 (44.2%)	
Enhancement pattern				
Typical	102 (65.8%)	74 (71.8%)	28 (53.8%)	**0.026**
Atypical	53 (34.2%)	29 (28.2%)	24 (46.2%)	
Delayed central enhancement				
Absent	135 (87.1%)	94 (91.3%)	41 (78.8%)	**0.029**
Present	20 (12.9%)	9 (8.7%)	11 (21.2%)	
Arterial peritumoral enhancement				
Absent	114 (73.5%)	85 (82.5%)	29 (55.8%)	**<0.001**
Present	41 (26.5%)	18 (17.5%)	23 (44.2%)	
Hepatobiliary phase hypointensity				
Atypical	68 (43.9%)	51 (49.5%)	17 (32.7%)	**0.046**
Typical	87 (56.1%)	52 (50.5%)	35 (67.3%)	
Peritumoral hypointensity on HBP				
Absent	103 (66.5%)	83 (80.6%)	20 (38.5%)	**<0.001**
Present	52 (33.5%)	20 (19.4%)	32 (61.5%)	

ER, early recurrence; BCLC, Barcelona Clinic Liver Cancer; HBV, hepatitis B virus; AFP, alpha-fetoprotein; PIVKA-II, protein induced by vitamin K absence or antagonist-II; GLOB, globulin; GGT, r-glutamyltransferase; AFU, a-fucosidase; PT, prothrombin time; CHOL, total cholesterol; MVI, microvascular invasion; APHE, arterial phase hyperenhancement; HBP, hepatobiliary phase. The value of p ≤ 0.05 is deepened in the table.

There were 155 patients [133 male patients (85.8%) and 22 female patients (14.2%)] with an average age of 55.41 ± 10.584 years. There were 52 patients in the early recurrence (ER) group and 103 patients in early recurrence free (ER-free) group. The positive rate of AFP-L3 (*p* = 0.013) in the ER group was higher than that in the ER-free group, and the other clinical and pathological factors were not statistically significant between the two groups.

Among the MRI features, the ER-free group had a higher probability of nonrim APHE (75.7% vs. 50.0%, *p* = 0.001) and atypical HBP hypointensity (49.5% vs. 32.7%, *p* = 0.046) than the ER group. Irregular shape (57.7% vs. 28.2%, *p <* 0.001), non-smooth margin (63.5% vs. 38.8%, *p* = 0.004), incomplete or absent radiological capsule enhancement (63.5% vs. 39.8%, 17.3% vs. 11.7%, *p* = 0.002), atypical enhancement pattern (46.2% vs. 28.2%, *p* = 0.026), rim APHE (36.5% vs. 20.4%, *p* = 0.030), delayed central enhancement (21.2% vs. 8.7%, *p* = 0.029), arterial peritumoral enhancement (44.2% vs. 17.5%, *p* < 0.001), and peritumoral hypointensity on HBP (61.5% vs. 19.4%, *p* < 0.001) had a higher probability in the ER group than in the ER-free group.

### Univariate and Multivariate Analysis Factors Predictive of ER

**Figure 2:**
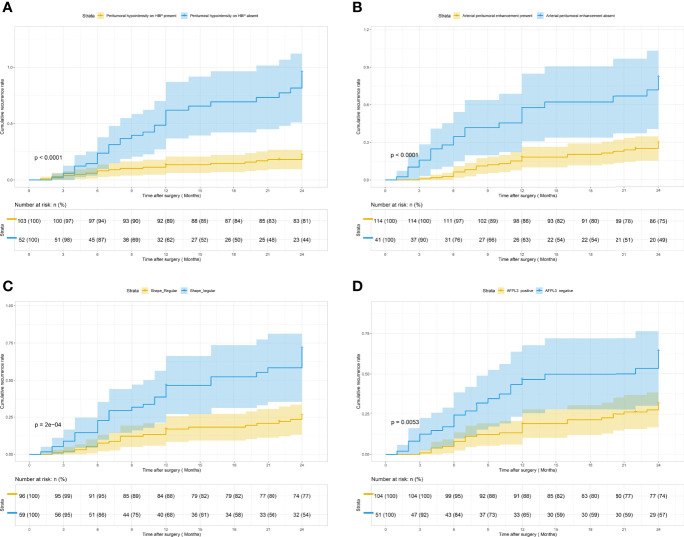
Kaplan–Meier curves for early recurrence showing that significant difference is seen between patients classified as follows: **(A)** peritumoral hypointensity on HBP (present) and peritumoral hypointensity on HBP (absent) (log-rank test, *p <*0.001); **(B)** arterial peritumoral enhancement (present) and arterial peritumoral enhancement (absent) (log-rank test, *p <*0.001); **(C)** shape (regular) and shape (irregular) (log-rank test, *p <*0.001); **(D)** AFP-L3 (positive) and AFP-L3 (negative) (log-rank test, *p* = 0.005).

In univariate analysis (**Table 2**), according to the test level of *p* < 0.05, a total of 10 features are related to early recurrence: shape (**Figure 2C**), margin, radiological capsule enhancement, nonrim APHE, rim APHE, enhancement pattern, delayed central enhancement, arterial peritumoral enhancement (**Figure 2B**), peritumoral hypointensity on HBP (**Figure 2A**), and AFP-L3 (**Figure 2D**). According to the variable conditions selected for multivariate Cox regression analysis, the test level of univariate analysis of radiological and pathological factors was *p* < 0.1, and the test level of clinical features was *p* < 0.3. Add 11 variables: MVI, microscopic capsule, MRI tumor diameter, nonperipheral “washout”, HBP hypointensity, AFP, PIVKAII, GLOB, GGT, AFU, and CHOL. The *p* = 0.304 of PT is at the critical value and does not meet the inclusion criteria. However, considering that it is the evaluation index of BCLC classification and related to liver function, it is included. Univariate analysis of all variables is shown in **Supplementary Table E4**.

**Table 2:** Univariate analysis for early recurrence.

Variable	HR	95% CI	*p*-value	Log rank
MVI	1.676	0.966, 2.906	0.066	0.060
Microscopic capsule	1.865	0.978, 3.558	0.059	0.052
MRI tumor diameter(cm)	1.149	0.990, 1.333	0.067	*NA*
Shape	2.699	1.556, 4.683	<0.001	<0.001
Margin	2.202	1.252, 3.873	0.006	0.004
Radiological capsule enhancement	1.750	1.191, 2.571	0.004	0.004
Nonrim APHE	2.570	1.491, 4.431	0.001	<0.001
Rim APHE	2.005	1.140, 3.528	0.016	0.013
Nonperipheral “washout”	1.644	0.951, 2.842	0.075	0.069
Enhancement pattern	1.889	1.094, 3.259	0.022	0.019
Delayed central enhancement	2.381	1.222, 4.639	0.011	0.008
Arterial peritumoral enhancement	2.923	1.689, 5.057	<0.001	<0.001
Hepatobiliary phase hypointensity	1.752	0.981, 3.129	0.058	0.052
Peritumoral hypointensity on HBP	4.163	2.374, 7.301	<0.001	<0.001
AFP-L3	2.118	1.227, 3.656	0.007	0.005
AFP (ng/L)	1.000	1.000, 1.001	0.214	*NA*
PIVKAII (mAU/ml)	1.000	1.000, 1.000	0.263	*NA*
GLOB (g/L)	0.945	0.889, 1.006	0.074	*NA*
GGT (U/L)	1.003	0.999, 1.007	0.174	*NA*
AFU (U/L)	1.020	0.990, 1.052	0.198	*NA*
PT (S)	0.858	0.640, 1.149	0.304	*NA*
CHOL (mmol/L)	0.781	0.529, 1.153	0.213	*NA*

p-value is the p-value of univariate Cox regression analysis; HR, hazard ratio; abbreviations can be found in the notes of **Table 1**.

All the above 22 variables were included in multivariate Cox regression analysis (forward LR), and six risk factors for predicting early recurrence of HCC were screened: shape (HR 2.326, 95% CI: 1.328–4.076, *p* = 0.003), arterial peritumoral enhancement (HR 2.282, 95% CI: 1.235–4.219, *p* = 0.008), AFP-L3 (HR 2.809, 95% CI: 1.577–5.005, *p* < 0.001), GGT (HR 1.006, 95% CI: 1.002–1.011, *p* = 0.008), PT (HR 0.720, 95% CI: 0.541–0.958, *p* = 0.024), and peritumoral hypointensity on HBP (HR 3.385, 95% CI: 1.859–6.163, *p* < 0.001). The above were independent risk factors except for PT and GGT. These six factors constitute the HBP-pre model because they do not contain postoperative pathological features and imaging features of HBP. The No HBP-pre model was composed of the remaining five risk factors excluding HBP imaging feature (peritumoral hypointensity on HBP). The postoperative pathological features of MVI and microscopic capsule (not independent risk factors) were added to the above two models to form a new HBP-post model and a new No HBP-post model. Multivariate Cox regression analysis for early recurrence can be found in **Table 3**.

**Table 3:** Multivariate Cox regression analysis for early recurrence.

	Preoperative Model					
	No HBP			HBP		
Variable	β	HR (95% CI)	*p*-value	β	HR (95% CI)	*p*-value
Shape (Irregular)	0.889	2.432 (1.388, 4.263)	0.002	0.844	2.326 (1.328, 4.076)	0.003
Arterial peritumoral enhancement*	1.143	3.135 (1.765, 5.570)	<0.001	0.825	2.282 (1.235, 4.219)	0.008
AFP-L3 (Positive)	0.856	2.354 (1.325, 4.182)	0.004	1.033	2.809 (1.577, 5.005)	<0.001
GGT (U/L)	0.005	1.005 (1.001, 1.010)	0.012	0.006	1.006 (1.002, 1.011)	0.008
PT (S)	−0.291	0.747 (0.563, 0.992)	0.044	−0.329	0.720 (0.541, 0.958)	0.024
Peritumoral hypointensity on HBP*	NA	NA	NA	1.219	3.385 (1.859, 6.163)	<0.001
	Postoperative Model					
	No HBP			HBP		
Variable	β	HR (95% CI)	*p*-value	β	HR (95% CI)	*p*-value
MVI*	0.309	1.362 (0.767, 2.418)	0.292	−0.047	0.954 (0.524, 1.738)	0.879
Microscopic capsule (Absent)	0.103	1.109 (0.553, 2.223)	0.772	0.310	1.363 (0.693, 2.681)	0.370
Shape (Irregular)	0.884	2.421 (1.368, 4.284)	0.002	0.813	2.254 (1.279, 3.971)	0.005
Arterial peritumoral enhancement*	1.081	2.948 (1.634, 5.317)	<0.001	0.796	2.217 (1.183, 4.153)	0.013
AFP-L3 (Positive)	0.840	2.315 (1.281, 4.184)	0.005	0.984	2.676 (1.487, 4.814)	0.001
GGT (U/L)	0.006	1.006 (1.001, 1.010)	0.009	0.006	1.006 (1.001, 1.011)	0.010
PT (S)	−0.295	0.745 (0.559, 0.993)	0.045	−0.312	0.732 (0.547, 0.979)	0.035
Peritumoral hypointensity on HBP*	NA	NA	NA	1.253	3.500 (1.881, 6.513)	<0.001

HR, hazard ratio; CI, confidence interval; MVI, microvascular invasion. * represents Present vs. Absent.

### Development and Validation of the Nomogram

**Figure 3:**
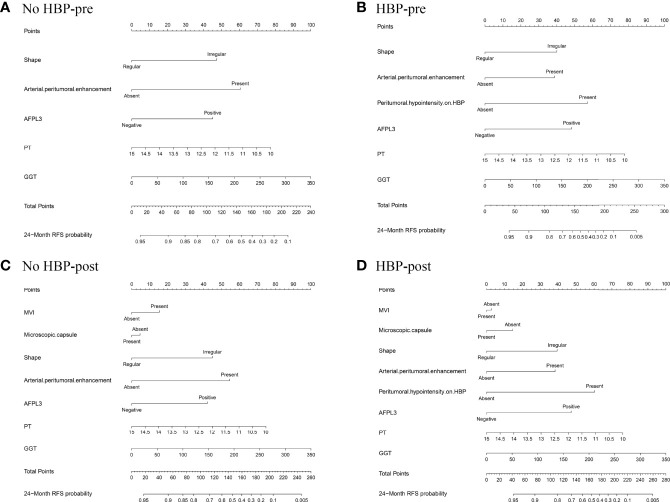
Nomograms predicting probability of early recurrence: **(A)** No HBP-pre model, the preoperative model without HBP imaging feature; **(B)** HBP-pre model, the preoperative model with HBP imaging feature; **(C)** No HBP-post model, the postoperative model without HBP imaging feature; **(D)** HBP-post model, the postoperative model with HBP imaging feature.

**Figure 4:**
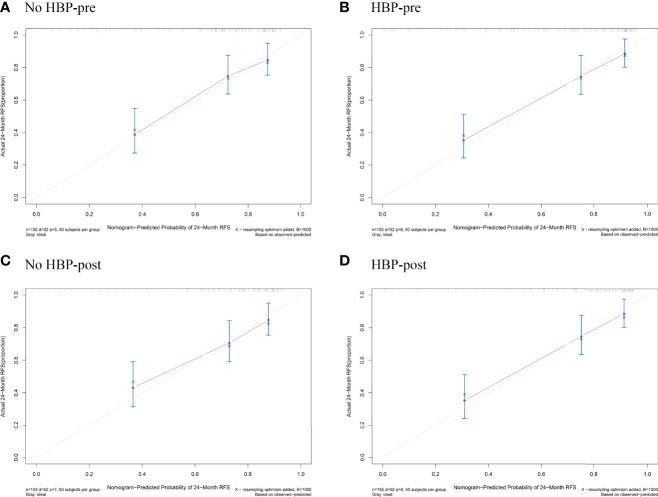
Calibration plots for the nomogram of prediction of RFS at 2 years using the No HBP-pre model **(A)**, the HBP-pre model **(B)**, the No HBP-post model **(C)**, and the HBP-post model **(D)**. RFS = recurrence-free survival.

The nomogram based on four models for predicting early recurrence of liver cancer is shown in **Figure 3**. The four nomograms have good discrimination and have little difference. The C-index of the No HBP-pre model and the HBP-pre model was 0.738 (95% CI: 0.664–0.813) and 0.766 (95% CI: 0.700–0.831), respectively. The C-index of the No HBP-post model and the HBP-post model was 0.742 (95% CI: 0.670–0.814) and 0.770 (95% CI: 0.706–0.833), respectively. The calibration curve (**Figure 4**) shows that the predicted RFS probability of the four nomograms is consistent with the estimated value of the actual RFS probability.

### Clinical Practicability of the Nomogram

**Figure 5:**
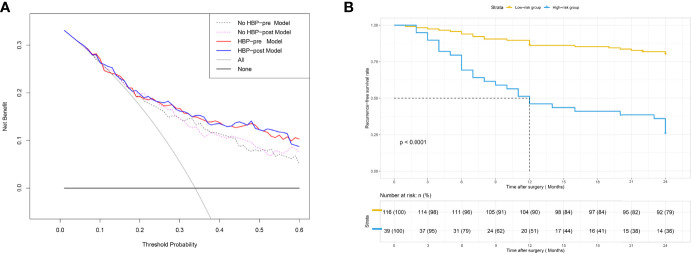
**(A)** DCA for four models. The use of the four nomograms for early recurrence prediction provides more benefit than two extreme conditions [the treat-all-patients scheme (gray line) and the treat-none scheme (horizontal black line)]. Nomograms of the HBP-pre model (red line) and the HBP-post model (blue line) received a higher net benefit than the model based on the No HBP-pre model (black dotted line) and the No HBP-post model (purple dotted line) when the threshold probability was greater than 25%. DCA = decision curve analysis. **(B)** Patients were stratified by the risk scores calculated with the HBP-pre model. The recurrence-free survival rate (RFS) of each group was calculated, and significant differences in each risk group were observed by the Log-rank test (*p* < 0.0001).

The DCA curve of the four nomograms is shown in **Figure 5A**. In DCA, when the threshold probability was greater than 15%, the net benefit of the four nomograms for early recurrence prediction was higher than the two extreme conditions. This indicated that the nomogram had potential clinical benefits. Nomograms of the HBP-pre model and the HBP-post model received a higher net benefit than the model based on the No HBP-pre model and the No HBP-post model when the threshold probability was greater than 25%. That indicated that the nomogram with HBP imaging feature had greater potential clinical benefits, which was independent preoperatively and postoperatively.

### Comparison Between Models

Because the C-index of the four models had little difference, ranging from 0.738 to 0.770, and the calibration curves had satisfactory prediction performance, we compared and analyzed the models and selected the optimal model to predict the early recurrence of HCC.

Comparison of postoperative and preoperative models: the NRI of the No HBP-post model vs. the No HBP-pre model and that of the HBP-post model vs. the HBP-pre model were 0.064 (95% CI: −0.193–0.369) and −0.040 (95% CI: −0.199–0.290), and IDI was 0.008 (95% CI: −0.005–0.069, *p* = 0.188) and 0.002 (95% CI: −0.006–0.043, *p* = 0.358), respectively. There was no statistical significance between the two models. It showed that the addition of postoperative pathological features had little significance to the model.

Comparison between models with or without HBP imaging feature: the NRI of the HBP-pre model vs. the No HBP-pre model and that of the HBP-post model vs. the No HBP-post model were 0.258 (95% CI: −0.001–0.508) and 0.280 (95% CI: 0.020–0.491), and the IDI was 0.092 (95% CI: 0.018–0.189, *p* = 0.014) and 0.086 (95% CI: 0.015–0.176, *p* = 0.008), respectively. Models (HBP-pre model and HBP-post model) with HBP imaging feature significantly improved the prediction efficiency. ANOVA compared the two models with statistical significance. It shows that the addition of HBP imaging feature was of the greatest significance to improve the diagnostic efficiency.

Therefore, we choose the HBP-pre model as the optimal model. Detailed parameters for comparison between models are shown in **Table 4**. The comparison figures between models are shown in **Supplementary Figure E1**.

**Table 4:** Comparison between models.

	*p*-value	C-index	NRI	IDI	*p*_1_-value
No HBP-pre Model	NA	0.738 (0.664, 0.813)	*NA*	*NA*	*NA*
HBP-pre Model*	<0.0001	0.766 (0.700, 0.831)	0.258 (−0.001, 0.508)	0.092 (0.018, 0.189)	0.014
No HBP-post Model*	0.5223	0.742 (0.670, 0.814)	0.064 (−0.193, 0.369)	0.008 (−0.005, 0.069)	0.188
HBP-post Model*	0.0005	0.770 (0.706, 0.833)	0.301 (0.056, 0.556)	0.095 (0.033, 0.198)	0.002
HBP-post vs. HBP-pre	0.6776	NA	−0.040 (−0.199, 0.290)	0.002 (−0.006, 0.043)	0.358
HBP-post vs. No HBP-post	<0.0001	NA	0.280 (0.020, 0.491)	0.086 (0.015, 0.176)	0.008

* represents that this model is compared with the No HBP-pre model. p-value is the probability when ANOVA compares the two models. p_1_-value is the probability of IDI calculation.

### HBP-Pre Model’s Ability to Evaluate Risk Stratification

**Figure 6:**
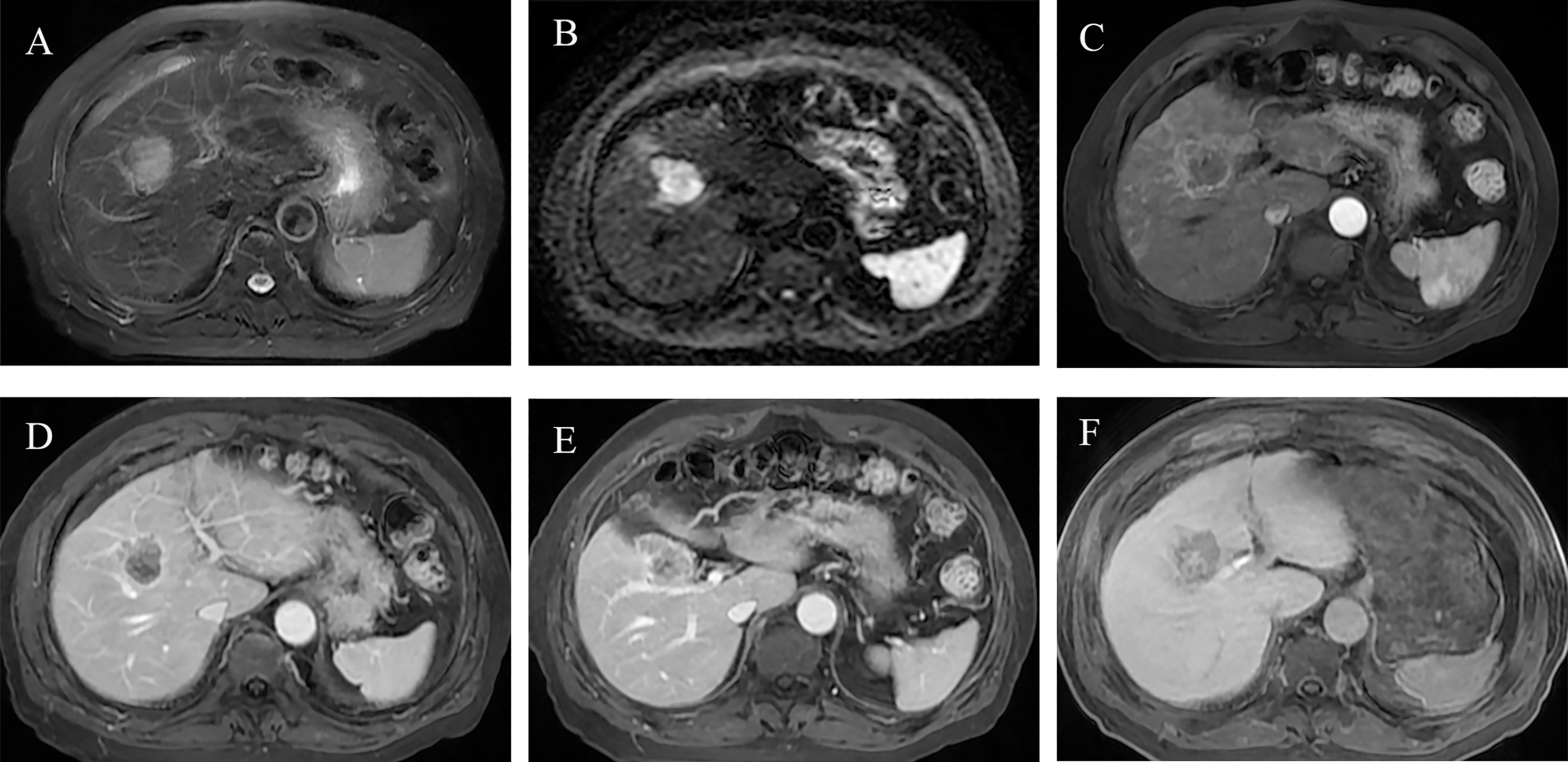
Preoperative MR images of early recurrence after anatomical hepatectomy in patients with hepatocellular carcinoma. A 72-year-old male with AFP-L3, PT, and GGT levels (negative, 10.7 s, 38 U/L) was hospitalized due to abdominal discomfort. Before operation, Gd-BOPTA MRI found a 3.7-cm lesion in hepatic segment V, with typical HCC features: mild hyperintensity on T2-weighted imaging **(A)**, hyperintensity on diffusion-weighted imaging **(B)**, and hypointensity on HBP **(F)**. Rim arterial phase hyperenhancement present **(C)** and incomplete radiological capsule enhancement **(E)** were risk factors of early recurrence in our study. Arterial peritumoral enhancement, an independent risk factor for early recurrence, was presented on arterial phase and portal phase **(C, D)**, white arrow). Peritumoral hypointensity on HBP (**F**, white arrow), also an independent risk factor. After anatomical hepatectomy, postoperative follow-up presented that this patient suffered from intrahepatic distant recurrence at 3 months.

The risk score of each patient was calculated by the HBP-pre model, ranging from −1.98 to 3.03. Patients were divided into two groups (low-risk group and high-risk group), taking 75% of the risk score distribution range as the dividing point (the cutoff score was 0.84806, the corresponding nomogram 24-month RFS probability is 0.47845). There were 116 patients in the low-risk group, of whom 23 patients had early recurrence, and the recurrence rate was 19.83%. There were 39 patients in the high-risk group, of whom 29 patients had early recurrence; the recurrence rate was 74.36%, and the median recurrence time was 12 months. RFS rate was observed in each risk group (*p* < 0.001, by the log-rank test) (**Figure 5B**). The ability of risk identification was proved by this model This model successfully predicted that a patient was at high-risk group, and its MRI was shown in **Figure 6**.

## Discussion

The purpose of our study was to establish a model for predicting early recurrence after anatomical hepatectomy of HCC based on the HBP imaging characteristics of gadobenate-enhanced MRI. We concluded that the HBP imaging features included in the model show better performance in predicting early recurrence compared with other models. Specifically, in this study, two models based on HBP imaging features of gadobenate-enhanced MRI provided better discrimination (C-index ≥ 0.766; *p*< 0.05), more accurate prediction (NRI ≥ 0.258, IDI ≥ 0.086), and better clinical utility.

This study also found that shape, arterial peritumoral enhancement, and peritumoral hypointensity on HBP in the gadobenate-enhanced MRI and AFP-L3 in clinical features were independent risk factors for predicting the early recurrence of HCC. These four independent risk factors were combined with GGT and PT to form an HBP-pre model. Based on this model, we established No HBP-pre, HBP-post, and No HBP-post models, respectively, by removing the imaging features of HBP and adding the postoperative pathological features of MVI and microscopic capsule (31–33). The discrimination degree and calibration curve of nomogram corresponding to the four models perform well, with little difference. However, through the comparison between models, we found that the accuracy and clinical benefit of models were not significantly improved after adding postoperative pathological features (MVI and microscopic capsule), but after adding the HBP imaging feature (peritumoral hypointensity on HBP), they were significantly improved. Considering the clinical practicability, we finally selected the HBP-pre model as the optimal model and its C-index reached 0.766. As far as we know, this is the first time that the specificity of gadobenate-enhanced MRI (with complete arterial phase, portal phase, DP, and HBP) is used to explore the role of HBP imaging features in predicting early recurrence. If confirmed in future research, we will pay attention to the role of HBP of gadobenate-enhanced MRI in predicting early recurrence of HCC after curative resection.

The arterial peritumoral enhancement and peritumoral hypointensity on HBP in the HBP-pre model were independent risk factors for predicting early recurrence, consistent with previous studies (10, 23, 34, 35), but refer to the peritumoral hypointensity on HBP of gadoxetic acid-enhanced MR. At the same time, these two features had also been proved by many studies to be a key factor in predicting MVI (14, 36), which is also considered to be one of the factors in predicting early recurrence (31). However, in our study, MVI has little correlation with early recurrence, and this may be related to our small sample size, which needs to be expanded for further research. Although shape is not a recognized predictor of early recurrence, it is similar to margin, which is a recognized factor (15, 34). Clinical features in the model, GGT is associated with early recurrence, which has been reported in previous studies (35), and PT has not been found. AFP is recognized as one of the factors that can predict early recurrence (10, 15), although it is not included in our model. AFP-L3, a subtype of AFP, which is rarely included in previous models, is included. Clinical studies have shown that AFP-L3 is a marker of the biological malignancy of liver cancer. HCC cells expressing AFP-L3 sugars tend to have early vascular infiltration and intrahepatic metastasis (37). Imaging studies showed that AFP-L3-positive HCC had abundant blood vessels, the blood supply came from hepatic artery, and the tumor doubling time was short (38). This suggests that AFP-L3-positive HCC has the potential for rapid growth and early distant metastasis (38, 39).

There is still the problem of high recurrence rate after hepatectomy. Although patients at higher risk of recurrence are potential candidates for most adjuvant clinical trials (3, 40, 41), there is no consensus that adjuvant therapy can reduce recurrence after resection. Primary liver transplantation is recognized as the most effective treatment to prevent recurrence (42), but the transplantation rate of HCC patients is very low. Even studies have proved that the survival outcome of salvage liver transplantation after hepatectomy is the same as that of primary liver transplantation of HCC (42, 43). Surgical resection followed by salvage liver transplantation is an alternative strategy for HCC patients at high risk of recurrence, and the transplantation rate is still very low (44, 45). Therefore, according to 90,600 new HCC patients worldwide in 2020, the proportion of liver transplantation is less than 20% (46), and the early recurrence rate was 32.9% (51/155) in our study, which was lower than that of other studies (10, 17, 44, 47). We tried to divide patients into low-, medium-, and high-risk groups according to the risk score distribution of the HBP-pre model, but the log rank test between the low-risk group and the medium-risk group was not statistically significant. Therefore, we chose 75% of the risk score distribution as the dividing point and divided it into the low-risk group and the high-risk group. There were 39 patients in the high-risk group and 29 patients with early recurrence, and the recurrence rate was as high as 74.36%. We recommend that primary or salvage liver transplantation should be given priority to patients with HCC in the high-risk group.

Our study has limitations. Firstly, this is a retrospective analysis and there may be selective offsets. For example, our findings cannot be applied to patients without HBP and patients with peritumoral artifacts on MR (patients with deteriorated liver function represent the unclear image of tumor margin). Secondly, the amount of data is small and the follow-up time is short because the Gd-BOPTA contrast agent was used in the hospital from 2016. Thirdly, because there are few hospitals using Gd-BOPTA hepatobiliary specific contrast agent, we did not conduct external validation. Because the early recurrence rate is low, we did not use the internal verification of dividing the data into a training and a validation cohort but used the internal verification of bootstrap 1,000 resampling to obtain more accurate and stable results. Finally, due to insufficient follow-up time, the predictors of late recurrence and survival were not studied. Follow-up and an in-depth study are thus necessary.

In conclusion, the HBP imaging features of gadobenate-enhanced MRI play an important role in predicting the early recurrence of HCC after curative resection. Eligible patients are recommended to perform HBP imaging scanning. The HBP-pre model is simple and convenient and can be used in clinical application after verification by follow-up studies.

## Data Availability Statement

The original contributions presented in the study are included in the article/**Supplementary Material**. Further inquiries can be directed to the corresponding authors.

## Ethics Statement

This study was approved by the Ethics Committee of Eastern Hepatobiliary Surgery Hospital, the Third Affiliated Hospital of Shanghai Naval Military Medical University, China, and waived the requirement of obtaining written informed consent.

## Author contributions

Study design: LWM, HL, and JNY. Data collection: LWM, SKR, ZW, HL, ZSS, and XXW. Data analysis: LWM and SKR. Writing: LWM and SKR. Revising: NJY and WPJ. All authors read and approved the file version of the manuscript.

## Funding

This work was partially supported by the National Nature Science Foundation of China (Grant Nos. 81830059).

## Conflict of Interest

The authors declare that the research was conducted in the absence of any commercial or financial relationships that could be construed as a potential conflict of interest.

## Publisher’s Note

All claims expressed in this article are solely those of the authors and do not necessarily represent those of their affiliated organizations, or those of the publisher, the editors and the reviewers. Any product that may be evaluated in this article, or claim that may be made by its manufacturer, is not guaranteed or endorsed by the publisher.
