# Supplementary material for: Hepatobiliary Phase Features of Preoperative Gadobenate-Enhanced MR can Predict Early Recurrence of Hepatocellular Carcinoma in Patients Who Underwent Anatomical Hepatectomy
Source: Front Oncol. 2022 Aug 3;12:862967. doi: 10.3389/fonc.2022.862967 (PMC9381876; doi:10.3389/fonc.2022.862967)
Supplement: Supplementary file 1 [file DataSheet_1.docx]

Table E1 Sequences and parameters of MRI

| **Sequences** | **Image plane** | **TR/TE (msec)** | **FOV (mm)** | **Flip angle** | **Thickness (mm)** | **Matrix** | **Scanning order** |
| --- | --- | --- | --- | --- | --- | --- | --- |
| FIESTA | C | 3.5/1.5 | 420 × 420 | 60 | 6 | 160 × 224 | 1 |
| T1WI | A | 190/4.3(2) | 420 × 420 | 80 | 6 | 256 × 160 | 3 |
| T2WI | A | 6667/85 | 420 × 420 | 160 | 6 | 320 × 224 | 4 |
| LAVA | A | 3.7/1.7 | 420 × 420 | 15 | 2.5 | 256 × 192 | 5 |
| 2D MRCP | Oblique | 4000/847 | 320 × 320 | 160 | 50 | 320 × 256 | 6 |
| HBP | A | 3.7/1.7 | 420 × 420 | 15 | 2.5 | 256 × 192 | 7 |
| HBP | C | 3.7/1.7 | 400 × 400 | 15 | 2.5 | 256 × 192 | 8 |

Table E2 The definition of LI-RADS imaging Features

| LI-RADS features | Definition |
| --- | --- |
| MRI Tumor diameter | Largest outer-edge-to-outer-edge dimension of an observation: Include “capsule” in measurement; Pick phase, sequence, plane in which margins are clearest; Do not measure in arterial phase or DWI if margins are clearly visible on different phase. |
| Blood products in mass | Intralesional or perilesional hemorrhage in the absence of biopsy, trauma or intervention. |
| Radiological capsule  enhancement | Enhancing “capsule”: Smooth, uniform, sharp border around most(incomplete) or all(complete) of an observation, unequivocally thicker or more conspicuous than fibrotic tissue around background nodules, and visible as enhancing rim in PVP, DP, or TP; Absent (Non enhancing “capsule”). Capsule appearance not visible as an enhancing rim. |
| Mild-moderate T2 hyperintensity | Intensity on T2WI mildly or moderately higher than liver and similar to or less than  non-iron-overloaded spleen. |
| Restricted diffusion | Intensity on DWI, not attributable solely to T2 shine-through, unequivocally higher than liver and/or ADC unequivocally lower than liver. |
| Nonrim APHE | Nonrim-like enhancement in arterial phase unequivocally greater in whole or in part than liver. Enhancing part must be higher in attenuation or intensity than liver in arterial phase. |
| Rim APHE | Spatially defined subtype of APHE in which arterial phase enhancement is most pronounced in observation periphery. |
| Nonperipheral "washout" | Nonperipheral visually assessed temporal reduction in enhancement in whole or in part relative to composite liver tissue from earlier to later phase resulting in hypoenhancement in the extracellular phase. |
| Hepatobiliary phase hypointensity | Intensity in the hepatobiliary phase unequivocally less, in whole or in part, than liver. |
| Delayed central enhancement | Spatially defined subtype of “washout” in which apparent washout is most pronounced in observation periphery |

**APHE, Arterial phase hyperenhancement; PVP, Portal venous phase; DP, Delayed phase.**

Table E3 Baseline patient characteristics

| **Characteristic** | **Total(n=155)** | **ER-free (n=103)** | **ER(n=52)** | ***p* value** |
| --- | --- | --- | --- | --- |
| **Clinical features** |  |  |  |  |
| Age | 55.41±10.584 | 55.55±10.117 | 55.12±11.552 | 0.809 |
| Sex |  |  |  |  |
| Male | 133(85.8%) | 90(87.4%) | 43(82.7%) | 0.430 |
| Female | 22(14.2%) | 13(12.6%) | 9(17.3%) |  |
| BCLC stage |  |  |  |  |
| 0 | 38(24.5%) | 29(28.2%) | 9(17.3%) | 0.330 |
| A | 104(67.1%) | 66(64.1%) | 38(73.1%) |  |
| B | 13(8.4%) | 8(7.8%) | 5(9.6%) |  |
| Child-Pugh stage |  |  |  |  |
| A | 148(95.5%) | 99(96.1%) | 49(94.2%) | 0.901 |
| B | 7(4.5%) | 4(3.9%) | 3(5.8%) |  |
| Liver disease |  |  |  |  |
| HBV | 139(89.7%) | 92(89.3%) | 47(90.4%) | 0.837 |
| None or other | 16(10.3%) | 11(10.7%) | 5(9.6%) |  |
| AFP-L3 |  |  |  |  |
| Negative | 104(67.1%) | 76(73.8%) | 28(53.8%) | **0.013** |
| Positive | 51(32.9%) | 27(26.2%) | 24(46.2%) |  |
| AFP(ng/L) | 25(4.4-175.7) | 16.8(4.5-189) | 44.2(3.6-167.2) | 0.417 |
| PIVKA-II(mAU/mL) | 119(30-916) | 119(27-883) | 120(47-1335.25) | 0.381 |
| CA199(U/mL) | 16.3(8.2-29.6) | 18.6(9.2-29.7) | 15.1(7.65-27.85) | 0.298 |
| CEA(ng/mL) | 2.3(1.6-3.3) | 2.4(1.6-3.2) | 2.15(1.4-3.4) | 0.641 |
| ALT(U/L) | 28(19-41) | 28(19-41) | 28.5(21.25-41) | 0.897 |
| AST(U/L) | 26(20-37) | 25(20-37) | 26.5(21-35.5) | 0.476 |
| TP(g/L) | 68.3(64.9-73.3) | 68(64.8-73.1) | 69.2(65.075-74.05) | 0.508 |
| ALB(g/L) | 42.8(39.6-46) | 42.7(39.6-45.5) | 43(39.825-46.15) | 0.597 |
| GLOB(g/L) | 26.2(23.2-29) | 26.75(23.875-26.75) | 25.2(22.4-25.2) | 0.056 |
| TBIL(μmol/L) | 14.4(11.9-18.5) | 14.4(11.7-18.6) | 14.4(12-18.2) | 0.915 |
| DBIL(μmol/L) | 5.4(4.2-7) | 5.4(4.2-7.2) | 5.5(4.25-6.875) | 0.931 |
| IBIL(μmol/L) | 8.8(7-11.6) | 9(7.1-11.4) | 8.7(6.85-11.95) | 0.776 |
| CHE(U/L) | 7434(6039-8349) | 7375(5947-8285) | 7559(6091.25-8600.5) | 0.476 |
| CG(ug/ML) | 1.2(0.5-2.5) | 1.2(0.5-2.3) | 1.1(0.5-2.575) | 0.729 |
| TBA(μmol/L) | 5.6(3.2-11.6) | 5.9(3.2-11.6) | 5.05(3.125-12.325) | 0.699 |
| GGT(U/L) | 40(26-73) | 39(25-63) | 43(28.5-102.25) | 0.212 |
| AFU(U/L) | 23(19-28) | 23(18-27) | 23.5(19.25-29.75) | 0.193 |
| CRP(mg/L) | 1.21(0.5-2.94) | 1.24(0.5-2.95) | 0.98(0.5-2.4875) | 0.330 |
| PLT(10^9/L) | 150.43±61.189 | 151.43±63.982 | 148.46±55.790 | 0.777 |
| PT(S) | 12(11.4-12.7) | 12.1(11.4-12.8) | 11.9(11.2-12.575) | 0.176 |
| APTT(S) | 28.6(26.2-32.2) | 28.9(26.3-32.3) | 28.45(25.1-31.75) | 0.262 |
| TT(S) | 20.3(19.4-21.1) | 20.3(19.4-21) | 20.4(19.2-21.3) | 0.934 |
| FBG(g/L) | 2.1(1.85-2.48) | 2.09(1.85-2.52) | 2.105(1.8375-2.4075) | 0.897 |
| CHOL(mmol/L) | 3.94(3.47-4.32) | 4.0234(3.55-4.38) | 3.8177(3.375-4.0825) | 0.155 |
| TG(mmol/L) | 1.0757(0.79-1.28) | 1.0757(0.79-1.28) | 1.1(0.805-1.26) | 0.847 |
| HDL(mmol/L) | 1.1967(1.04-1.39) | 1.23(1.04-1.39) | 1.1934(1.0125-1.3975) | 0.393 |
| LDL(mmol/L) | 2.51(2.03-2.84) | 2.5537(2.03-2.91) | 2.3958(2-2.6775) | 0.214 |
| HBsAg |  |  |  |  |
| Negative | 22(14.2%) | 15(14.6%) | 7(13.5%) | 0.853 |
| Positive | 133(85.8%) | 88(85.4%) | 45(86.5%) |  |
| HBsAb |  |  |  |  |
| Negative | 136(87.7%) | 90(87.4%) | 46(88.5%) | 0.846 |
| Positive | 19(12.3%) | 13(12.6%) | 6(11.5%) |  |
| HBeAg |  |  |  |  |
| Negative | 116(74.8%) | 79(76.7%) | 37(71.2%) | 0.453 |
| Positive | 39(25.2%) | 24(23.3%) | 15(28.8%) |  |
| HBeAb |  |  |  |  |
| Negative | 54(34.8%) | 34(33.0%) | 20(38.5%) | 0.501 |
| Positive | 101(65.2%) | 69(67.0%) | 32(61.5%) |  |
| HBcAb |  |  |  |  |
| Negative | 4(2.6%) | 1(1.0%) | 3(5.8%) | 0.214 |
| Positive | 151(97.4%) | 102(99%) | 49(94.2%) |  |
| HBV/C-DNA |  |  |  |  |
| <50IU/ml | 70(45.2%) | 44(42.7%) | 26(50.0%) | 0.539 |
| 50-10^3 | 22(14.2%) | 14(13.6%) | 8(15.4%) |  |
| 10^3-10^5 | 31(20.0%) | 24(23.3%) | 7(13.5%) |  |
| >10^5 | 32(20.6%) | 21(20.4%) | 11(21.2%) |  |
| **Pathologic factors** |  |  |  |  |
| MVI |  |  |  |  |
| Absent | 105(67.7%) | 75(72.8%) | 30(58.8%) | 0.057 |
| Present | 50(32.3%) | 28(27.2%) | 22(42.3%) |  |
| Surgical capsule |  |  |  |  |
| Present | 95(61.3%) | 63(61.2%) | 32(61.5%) | 0.964 |
| Absent | 60(38.7%) | 40(38.8%) | 20(38.5%) |  |
| Microscopic capsule |  |  |  |  |
| Present | 130(83.9%) | 90(87.4%) | 40(76.9%) | 0.095 |
| Absent | 25(16.1%) | 13(12.6%) | 12(23.1%) |  |
| Microscopic cirrhosis |  |  |  |  |
| Absent | 100(64.5%) | 68(66.0%) | 32(61.5%) | 0.582 |
| Present | 55(35.5%) | 35(34.0%) | 20(38.5%) |  |
| Microscopic small foci of cancer adjacent | |  |  |  |
| Absent | 68(43.9%) | 49(47.6%) | 19(36.5%) | 0.191 |
| Present | 87(56.1%) | 54(52.4%) | 33(63.5%) |  |
| Satellite nodules |  |  |  |  |
| Absent | 138(89%) | 93(90.3%) | 45(86.5%) | 0.480 |
| Present | 17(11%) | 10(9.7%) | 7(13.5%) |  |
| Edmondson-Steiner grade | |  |  |  |
| I-II | 22(14.2%) | 16(15.5%) | 6(11.5%) | 0.501 |
| III-IV | 133(85.8%) | 87(84.5%) | 46(88.5%) |  |
| **MRI features** |  |  |  |  |
| MRI Tumor diameter(cm) | 3(2.1-4.3) | 2.9(2.1-4.1) | 3.1(2.2-5.175) | 0.221 |
| Tumor number |  |  |  |  |
| Solitary | 138(89%) | 94(91.3%) | 44(84.6%) | 0.211 |
| Multiple | 17(11%) | 9(8.7%) | 8(15.4%) |  |
| Shape |  |  |  |  |
| Regular | 96(61.9%) | 74(71.8%) | 22(42.3%) | **<0.001** |
| Irregular | 59(38.1%) | 29(28.2%) | 30(57.7%) |  |
| Margin |  |  |  |  |
| Smooth | 82(52.9%) | 63(61.2%) | 19(36.5%) | **0.004** |
| Non-smooth | 73(47.1%) | 40(38.8%) | 33(63.5%) |  |
| Intratumoral necrosis |  |  |  |  |
| Absent | 120(77.4%) | 83(80.6%) | 37(71.2%) | 0.185 |
| Present | 35(22.6%) | 20(19.4%) | 15(28.8%) |  |
| Blood products in mass |  |  |  |  |
| Absent | 141(91.0%) | 94(91.3%) | 47(90.4%) | 0.857 |
| Present | 14(9.0%) | 9(8.7%) | 5(9.6%) |  |
| Radiological capsule enhancement | |  |  |  |
| Complete | 60(38.7%) | 50(48.5%) | 10(19.2%) | **0.002** |
| Incomplete | 74(47.7%) | 41(39.8%) | 33(63.5%) |  |
| Absent | 21(13.5%) | 12(11.7%) | 9(17.3%) |  |
| Mild-moderate T2 hyperintensity | |  |  |  |
| Absent | 36(23.2%) | 21(20.4%) | 15(28.8%) | 0.239 |
| Present | 119(76.8%) | 82(79.6%) | 37(71.2%) |  |
| Restricted diffusion |  |  |  |  |
| Present | 143(92.3%) | 93(90.3%) | 50(96.2%) | 0.197 |
| Absent | 12(7.7%) | 10(9.7%) | 2(3.8%) |  |
| Nonrim APHE |  |  |  |  |
| Present | 104(67.1%) | 78(75.7%) | 26(50.0%) | **0.001** |
| Absent | 51(32.9%) | 25(24.3%) | 26(50.0%) |  |
| Rim APHE |  |  |  |  |
| Absent | 115(74.2%) | 82(79.6%) | 33(63.5%) | **0.030** |
| Present | 40(25.8%) | 21(20.4%) | 19(36.5%) |  |
| Nonperipheral"washout" |  |  |  |  |
| Present | 101(65.2%) | 72(69.9%) | 29(55.8%) | 0.081 |
| Absent | 54(34.8%) | 31(30.1%) | 23(44.2%) |  |
| Enhancement pattern |  |  |  |  |
| Typical | 102(65.8%) | 74(71.8%) | 28(53.8%) | **0.026** |
| Atypical | 53(34.2%) | 29(28.2%) | 24(46.2%) |  |
| Delayed central enhancement | |  |  |  |
| Absent | 135(87.1%) | 94(91.3%) | 41(78.8%) | **0.029** |
| Present | 20(12.9%) | 9(8.7%) | 11(21.2%) |  |
| Arterial peritumoral enhancement | |  |  |  |
| Absent | 114(73.5%) | 85(82.5%) | 29(55.8%) | **<0.001** |
| Present | 41(26.5%) | 18(17.5%) | 23(44.2%) |  |
| Hepatobiliary phase hypointensity | |  |  |  |
| Atypical | 68(43.9%) | 51(49.5%) | 17(32.7%) | **0.046** |
| Typical | 87(56.1%) | 52(50.5%) | 35(67.3%) |  |
| Peritumoral hypointensity on HBP | |  |  |  |
| Absent | 103(66.5%) | 83(80.6%) | 20(38.5%) | **<0.001** |
| Present | 52(33.5%) | 20(19.4%) | 32(61.5%) |  |
| MRI Liver cirrhosis |  |  |  |  |
| Absent | 41(26.5%) | 29(28.2%) | 12(23.1%) | 0.499 |
| Present | 114(73.5%) | 74(71.8%) | 40(76.9%) |  |
| Splenomegaly |  |  |  |  |
| Absent | 87(56.1%) | 58(56.3%) | 29(55.8%) | 0.949 |
| Present | 68(43.9%) | 45(43.7%) | 23(44.2%) |  |
| Ascites |  |  |  |  |
| Absent | 149(96.1%) | 98(95.1%) | 51(98.1%) | 0.372 |
| Present | 6(3.9%) | 5(4.9%) | 1(1.9%) |  |

**ER, early recurrence; BCLC, Barcelona Clinic Liver Cancer; HBV, hepatitis B virus; MVI, microvascular invasion；APHE,** **arterial phase hyperenhancement; HBP, hepatobiliary phase; AFP, alpha-fetoprotein; PIVKA-II, protein induced by vitamin K absence or antagonist-II; GLOB, globulin; GGT, r-glutamyltransferase; AFU,** **a-fucosidase; PT, prothrombin time; CHOL, total cholesterol; CA199,** **carbohydrate antigen 19-9; CEA,** **carcinoembryonic antigen; ALT,** **alanine aminotransferase; AST, aspartate aminotransaminase; TP, total protein; TBIL, total bilirubin; DBIL, direct bilirubin; IBIL,** **indirect bilirubin; CHE, cholinesterase; CG, glycocholic acid; TBA, total bile acid; CRP,** **C-reactive protein; PLT, platelet count; APTT, activated partial thromboplastin time; TT, thrombin time; FBG, fibrinogen; TG, triglyceride; HDL, high density lipoprotein; LDL-C, low density lipoprotein.**

Table E4 Univariate analysis for early recurrence

| Variable | HR | 95%CI | *P* Value | Log Rank |
| --- | --- | --- | --- | --- |
| Age | 0.997 | 0.971,1.024 | 0.845 | *NA* |
| Sex | 1.439 | 0.701,2.952 | 0.321 | 0.313 |
| BCLC stage | 1.417 | 0.865,2.323 | 0.167 | 0.335 |
| Child-Pugh stage | 0.572 | 0.178,1.837 | 0.348 | 0.337 |
| Liver disease | 0.977 | 0.389,2.458 | 0.961 | 0.961 |
| MVI | 1.676 | 0.966,2.906 | 0.066 | 0.060 |
| Surgical capsule | 1.019 | 0.583,1.782 | 0.947 | 0.946 |
| Microscopic capsule | 1.865 | 0.978,3.558 | 0.059 | 0.052 |
| Microscopic cirrhosis | 1.162 | 0.664,2.031 | 0.600 | 0.595 |
| Microscopic small foci of cancer adjacent | 1.408 | 0.801,2.477 | 0.234 | 0.227 |
| Satellite nodules | 1.236 | 0.558,2.742 | 0.602 | 0.597 |
| Edmondson-Steiner grade | 1.392 | 0.594,3.259 | 0.446 | 0.439 |
| MRI Tumor diameter(cm） | 1.149 | 0.990,1.333 | 0.067 | *NA* |
| Tumor number | 1.742 | 0.820,3.703 | 0.149 | 0.139 |
| Shape | 2.699 | 1.556,4.683 | <0.001 | <0.001 |
| Margin | 2.202 | 1.252,3.873 | 0.006 | 0.004 |
| Intratumoral necrosis | 1.528 | 0.839,2.785 | 0.166 | 0.158 |
| Blood products in mass | 1.093 | 0.435,2.749 | 0.849 | 0.848 |
| Radiological capsule enhancement | 1.750 | 1.191,2.571 | 0.004 | 0.004 |
| Mild-moderate T2 hyperintensity | 0.673 | 0.369,1.226 | 0.196 | 0.188 |
| Restricted diffusion | 0.397 | 0.097,1.632 | 0.200 | 0.180 |
| Nonrim APHE | 2.570 | 1.491,4.431 | 0.001 | <0.001 |
| Rim APHE | 2.005 | 1.140,3.528 | 0.016 | 0.013 |
| Nonperipheral"washout" | 1.644 | 0.951,2.842 | 0.075 | 0.069 |
| Enhancement pattern | 1.889 | 1.094,3.259 | 0.022 | 0.019 |
| Delayed central enhancement | 2.381 | 1.222,4.639 | 0.011 | 0.008 |
| Arterial peritumoral enhancement | 2.923 | 1.689,5.057 | <0.001 | <0.001 |
| Hepatobiliary phase hypointensity | 1.752 | 0.981,3.129 | 0.058 | 0.052 |
| Peritumoral hypointensity on HBP | 4.163 | 2.374,7.301 | <0.001 | <0.001 |
| MRI_Liver cirrhosis | 1.186 | 0.622,2.261 | 0.604 | 0.600 |
| Splenomegaly | 1.029 | 0.595,1.779 | 0.918 | 0.917 |
| Ascites | 0.434 | 0.060,3.139 | 0.408 | 0.389 |
| AFP-L3 | 2.118 | 1.227,3.656 | 0.007 | 0.005 |
| AFP(ng/L) | 1.000 | 1.000,1.001 | 0.214 | *NA* |
| PIVKAII(mAU/mL) | 1.000 | 1.000,1.000 | 0.263 | *NA* |
| CA199(U/mL) | 0.996 | 0.981,1.011 | 0.592 | *NA* |
| CEA(ng/mL) | 1.001 | 0.820,1.222 | 0.993 | *NA* |
| ALT(U/L) | 0.998 | 0.989,1.006 | 0.583 | *NA* |
| AST(U/L) | 1.001 | 0.998,1.004 | 0.428 | *NA* |
| TP(g/L) | 1.014 | 0.972,1.058 | 0.519 | *NA* |
| ALB(g/L) | 0.997 | 0.957,1.038 | 0.882 | *NA* |
| GLOB(g/L) | 0.945 | 0.889,1.006 | 0.074 | *NA* |
| TBIL(μmol/L) | 0.995 | 0.949,1.043 | 0.839 | *NA* |
| DBIL(μmol/L) | 0.970 | 0.863,1.091 | 0.615 | *NA* |
| IBIL(μmol/L) | 1.003 | 0.933,1.077 | 0.943 | *NA* |
| CHE(U/L) | 1.000 | 1.000,1.000 | 0.498 | *NA* |
| CG(ug/ML) | 0.988 | 0.933,1.046 | 0.670 | *NA* |
| TBA(μmol/L) | 0.993 | 0.962,1.024 | 0.636 | *NA* |
| GGT(U/L) | 1.003 | 0.999,1.007 | 0.174 | *NA* |
| AFU(U/L) | 1.020 | 0.990,1.052 | 0.198 | *NA* |
| CRP(mg/L) | 0.989 | 0.963,1.016 | 0.437 | *NA* |
| PLT(10^9/L) | 0.999 | 0.995,1.004 | 0.726 | *NA* |
| PT(S） | 0.858 | 0.64,1.149 | 0.304 | *NA* |
| APTT(S) | 0.982 | 0.925,1.043 | 0.564 | *NA* |
| TT(S) | 1.057 | 0.875,1.276 | 0.568 | *NA* |
| FBG(g/L) | 1.179 | 0.781,1.781 | 0.433 | *NA* |
| CHOL(mmol/L) | 0.781 | 0.529,1.153 | 0.213 | *NA* |
| TG(mmol/L) | 1.371 | 0.694,2.710 | 0.364 | *NA* |
| HDLC(mmol/L) | 0.795 | 0.349,1.810 | 0.585 | *NA* |
| LDLC(mmol/L) | 0.803 | 0.524,1.231 | 0.315 | *NA* |
| HBsAg | 1.021 | 0.460,2.264 | 0.960 | 0.959 |
| HBsAb | 0.898 | 0.383,2.102 | 0.804 | 0.801 |
| HBeAg | 1.306 | 0.717,2.379 | 0.384 | 0.377 |
| HBeAb | 0.797 | 0.456,1.393 | 0.426 | 0.420 |
| HBcAb | 0.269 | 0.084,0.866 | 0.028 | 0.017 |
| HBV/C-DNA | 0.907 | 0.720,1.143 | 0.408 | 0.600 |

***P* Value is the *p* value of univariate Cox regression analysis; HR, Hazard Ratio;**

**Abbreviations can be found in the notes of Table E3.**


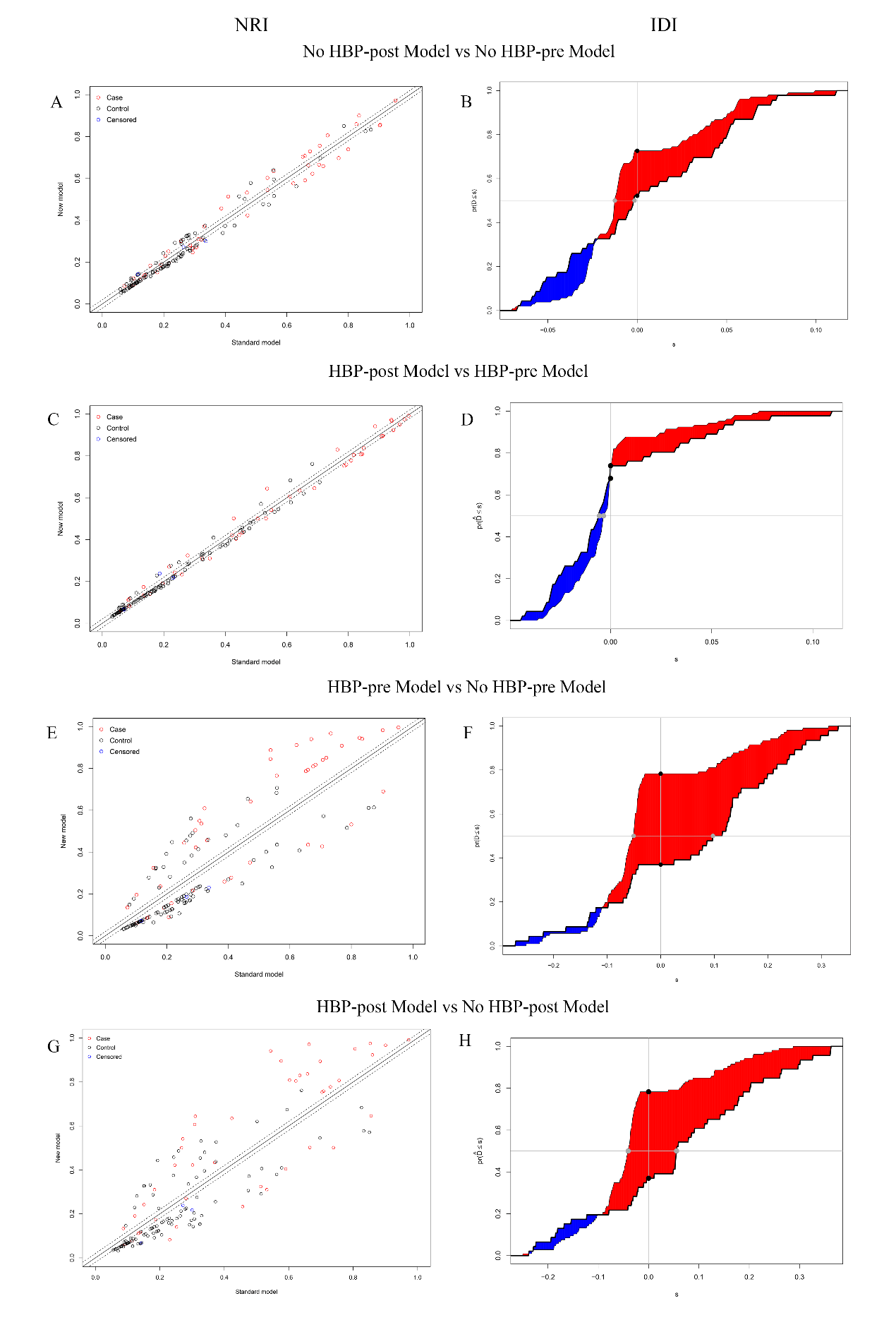


**FigureE1**

**NRI and IDI diagram of comparison between models.**
